# Method of Optimisation for Ambient Temperature Cured Sustainable Geopolymers for 3D Printing Construction Applications

**DOI:** 10.3390/ma12060902

**Published:** 2019-03-18

**Authors:** Shin Hau Bong, Behzad Nematollahi, Ali Nazari, Ming Xia, Jay Sanjayan

**Affiliations:** Centre for Sustainable Infrastructure, Faculty of Science, Engineering and Technology, Swinburne University of Technology, 3122 Melbourne, Australia; alinazari@swin.edu.au (A.N.); mxia@swin.edu.au (M.X.); jsanjayan@swin.edu.au (J.S.)

**Keywords:** 3D-concrete-printing, ambient temperature curing, shape retention ability, extrudability, mechanical properties, geopolymer

## Abstract

Since the initial introduction of geopolymers, these materials have been characterised as environmentally-friendly sustainable substitutes for ordinary Portland cement (OPC). There is a routine increase in the application of geopolymers, especially in advanced technologies. Because of its better rheological characteristics compared to OPC, geopolymers are appropriate materials for extrusion-based 3D printing technologies. This paper focuses on the optimisation of an ambient temperature cured geopolymer for 3D printing construction applications. The effects of mixture parameters, including the type of hydroxide solution (HS), the type of silicate solution (SS) and the mass ratio of SS to HS on the workability, extrudability, shape retention ability and mechanical performance of different geopolymer mixtures were investigated. Accordingly, an optimum mixture was identified for geopolymers cured at ambient temperatures. Mechanical properties of the optimised mixture, including flexural and compressive strengths, were measured in different directions with respect to the printed layers. Further, uniaxial tension tests were also conducted on the optimised mixture to measure its interlayer bond strength. The results showed that among the activators investigated, the sodium-based activator composed of sodium hydroxide and sodium silicate solutions, with a SiO_2_/Na_2_O ratio of 3.22, was the most effective activator, providing appropriate workability and extrudability, along with reasonable strength and a high shape retention ability. The acquired mechanical properties exhibited anisotropic behaviour in different testing direction. The strength of the interlayer bond was found to be adequate to avoid interfacial shear failure.

## 1. Introduction

Additive manufacturing (AM), or 3D printing in other terms, utilises an automated assembly procedure which manufactures layered components from digital model data. Despite the extensive application of AM in advanced areas of science such as the biomedical, aerospace and automotive fields [1], its development in the construction industry is still in its infancy. In the past few years, the application of AM in the construction industry has attracted a significant amount of attention. This is because AM can offer several benefits when compared to the conventional construction approach, such as improved geometrical freedom, increased safety in construction and reduced construction time, cost and waste [2,3]. There are a wide variety of challenges to be addressed before AM technologies could be thoroughly implemented in the construction industry. Some of the challenges include: (1) Limited scope of 3D printable cementitious materials, (2) limited reinforcing methods for 3D printed building components, (3) the existing design approaches and standards are not applicable for automated construction and (4) the high cost of construction-scale 3D-concrete-printing (3DCP) machines [4,5]. 

In recent years, extrusion-based and powder-based AM techniques, which are the primary methods of approximately all AM procedures, have been used to develop several 3DCP technologies. The powder particles are selectively bonded together by use of a liquid binder in the powder-based 3DCP [2]. Examples of powder-based 3DCP technologies are Emerging Objects [6] and D-shape [7]. In extrusion-based 3DCP, the cementitious material is extruded from a nozzle mounted on a robotic arm, crane or gantry. Examples of extrusion-based 3DCP technologies are Contour Crafting [8,9,10], Concrete Printing [11,12] and CONPrint3D [13].

Most of the printable cementitious materials used in extrusion-based 3DCP techniques are ordinary Portland cement (OPC)-based binders [11,12,14,15]. The production of OPC emits a significant amount of carbon dioxide (CO_2_) into the atmosphere [16]. In addition, the manufacture of OPC is significantly energy intensive [17]. Both of these factors may compromise the sustainability of 3DCP using OPC-based materials. Hence, it is necessary to develop materials without OPC that are appropriate for extrusion-based 3DCP processes. Geopolymer, a cement-free alternative binder to OPC, is the resultant of a geopolymerisation process where an aluminosilicate resource, such as fly ash or blast furnace slag, is activated by an alkali solution [18]. Geopolymers are affordable environmentally friendly products, where the energy consumption and carbon dioxide emission for the production of fly ash-based geopolymer, for example, is 60% and 80% less than OPC, respectively [19,20]. Apart from being environmentally friendly, geopolymers are specifically suitable for extrusion-based printing because they have modifiable setting characteristics [21] and gain higher strength much faster than OPC. 

Panda et al. [22] examined a geopolymer mix suitable for extrusion-based 3DCP. Panda et al. [23] have also investigated the effect of aluminosilicate resources such as silica fume, fly ash and slag on the rheological and hardened properties of printable geopolymer mixtures. However, the effects of alkaline activators on the printability of geopolymer have not yet been investigated. Results of previous studies have indicated that the hydroxide solution (HS), silicate solution (SS) and the mass ratio of SS to HS not only affect properties of hardened geopolymer, but also significantly influence the rheological properties of fresh geopolymer [24,25,26,27,28]. In the extrusion-based 3DCP, the rheology of fresh concrete is a critical parameter that extensively affects the fresh properties of printable concrete, including open time, workability, extrudability and buildability [11,29]. Therefore, this paper investigates the effects of several parameters, including the type of HS, the type of SS and the mass ratio of SS to HS on workability, shape retention ability, extrudability and mechanical properties of printable geopolymer mixtures. The final aim is to identify a 3D printable geopolymer mixture which has the optimum characteristics and is curable at ambient temperature. A series of tests were conducted to characterise the mechanical properties of the optimised mixture, including the compressive strength, flexural strength and interlayer bond strength. 

## 2. Materials

Fly ash and granulated ground blast furnace slag (henceforth referred to slag) were used as the source materials to synthesise the 3D printable geopolymer mixtures in this paper. The fly ash was supplied from Gladstone power station, Gladstone, Australia and classified as a low calcium (Class F) fly ash. The slag was supplied by Building Products Supplies Pty Ltd., Melbourne, Australia. The chemical composition of the fly ash and slag were determined by X-ray fluorescence (XRF, SPECTRO Analytical Instruments Inc., Mahwah, NJ, USA) analysis, and the results are presented in Table 1. The total percentages do not sum to 100% due to rounding. 

**Figure 1:**
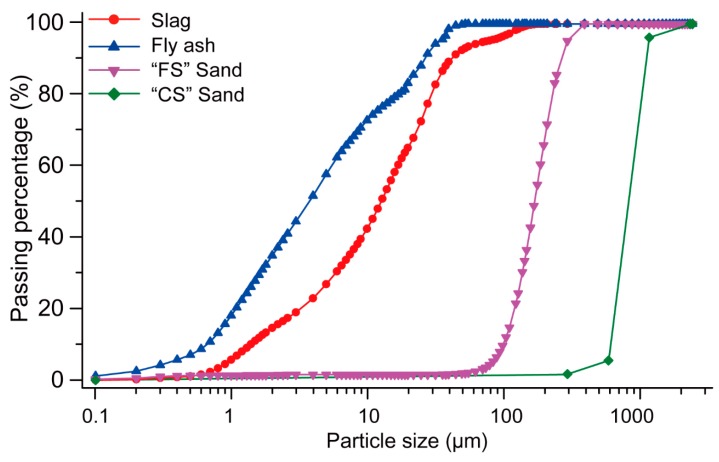
Particle size distributions of the fly ash, slag, fine sand (FS) and coarser sand (CS).

Two different sizes of silica sands were used in this paper. The fine sand supplied by TGS Industrial Sands Pty Ltd., Melbourne, Australia was denoted as “FS” and had a D50 of 172 µm. The relatively coarser sand, supplied by Sibelco Australia Ltd. (Melbourne, Australia), was denoted as “CS” and had a D50 of 898 µm. The particle size distributions of the fly ash, slag and both silica sands are shown in Figure 1.

Two types of sodium silicate (Na_2_SiO_3_) solutions and two types of potassium silicate (K_2_SiO_3_) solutions were used as silicate solutions (SSs) in this paper. All SSs were supplied by PQ Australia Pty Ltd. (Melbourne, Australia) The specifications of the SSs are presented in Table 2. Two types of HSs (a sodium hydroxide (NaOH) solution and a potassium hydroxide (KOH) solution) were also used in this paper. Both HSs were supplied by Sigma Aldrich Pty Ltd., Sydney, Australia. The NaOH and KOH pellets were dissolved in tap water to produce NaOH and KOH solutions with 8.0 M concentration. Both hydroxide solutions were left to cool in the laboratory environment before mixing with the silicate solutions with different SS/HS mass ratios. 

Four different activator solutions, including two sodium (Na)-based and two potassium (K)-based solutions, were designed by mixing HS and SS with different mass ratios. The Na-based activators were: (1) A combination of NaOH and D grade Na_2_SiO_3_ solutions and (2) a combination of NaOH and N grade Na_2_SiO_3_ solutions. The K-based activators were: (1) A combination of KOH and KASIL 2040 grade K_2_SiO_3_ solutions and (2) a combination of KOH and KASIL 2236 grade K_2_SiO_3_ solutions. In both Na-based and K-based activators, three different mass ratios of SS to HS were used: 1.5, 2.5 and 3.0.

Admixtures such as anhydrous borax and sodium carboxymethyl cellulose (CMC) powders were used as retarder and viscosity modifying agents, respectively.

## 3. Experimental Procedure

The procedure followed in this paper can be separated into two parts:

In part I, 12 mixtures were designed and the effects of different parameters, including the type of HS, the type of SS and the mass ratio of SS to HS, on workability, extrudability, compressive strength and shape retention ability of printed mixtures were investigated. All specimens printed in this part were consisted of only a single layer. An optimum mixture exhibiting desirable properties was selected based on the results obtained in part I.

In part II, two-layer specimens were printed using the optimum mixture. The flexural and compressive strengths of the samples, in different load directions, with respect to the printed layers, were measured. Interlayer bond strength was the other feature investigated in part II. 

### 3.1. Mixture Proportions and Mixing Procedure

Table 3 represents mixtures proportions of the 12 designed mixtures. In all mixtures, the mass ratio of fly ash to slag was kept constantly equal to 3.0. The mass ratio of activator to geopolymer source materials (fly ash and slag) was also kept constantly equal to 0.4. The dosages of anhydrous borax and CMC powders in each mixture were adjusted to achieve the appropriate rheological properties suitable for extrusion-based 3DCP (visually assessed). The water-to-geopolymer-solids ratio (W/GP-solids) of each mix [30] was also given in Table 3.

The fly ash, slag, anhydrous borax and sands were added to a Hobart mixer (Hobartcorp, Troy, OH, USA) and dry-mixed at low-speed for 3 min. The activator solutions were then gradually added to the mixer and mixing was continued for another 7 min. Once the materials were fully mixed and a uniform mixture was obtained, the CMC powder was added to the mixer and the mixing was continued for another 5 min to obtain the appropriate rheology for extrusion. 

### 3.2. Printing and Curing of Specimens

**Figure 2:**
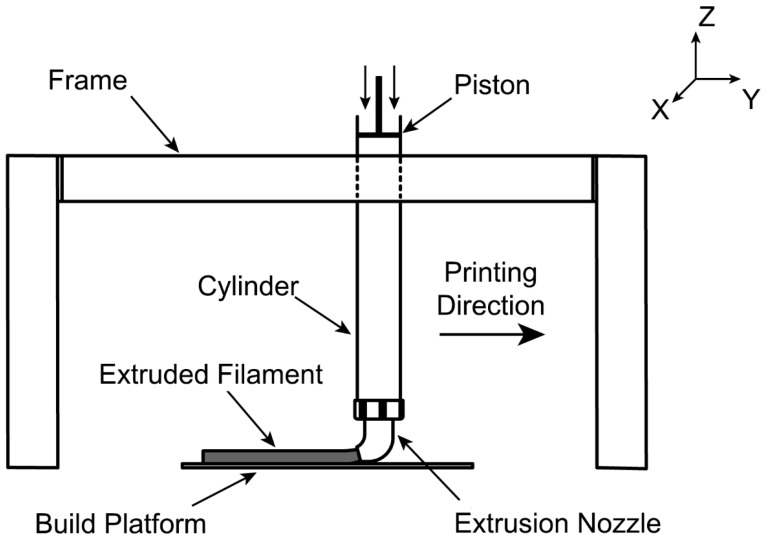
Schematic drawing of the custom-made 3D printing apparatus.

**Figure 3:**
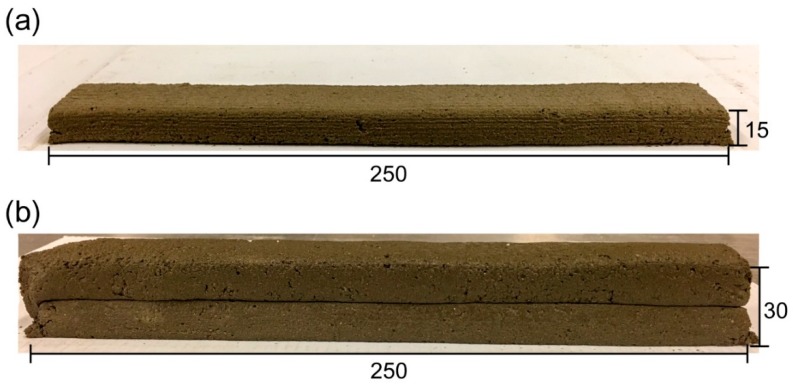
(**a**) One layer of 3D printed geopolymer filament. (**b**) Two layers of 3D printed geopolymer filament.

A custom-made small-scale 3D printing apparatus (designed and manufactured by the authors) was used for the printing process (Figure 2). The apparatus has a piston-type extruder, where fresh material was extruded from a 45° extrusion nozzle with a rectangle opening (30 mm × 15 mm). An external vibration was applied to the extruder while loading the fresh mixture to ensure the mixture inside the extruder received adequate compaction. The specimens were printed by moving the extruder in a horizontal direction at a constant speed. In part I, one-layer filaments with dimensions of 250 mm × 30 mm × 15 mm (L × W × H) were extruded for each mixture (Figure 3a). In part II, two-layer filaments with the dimensions of 250 mm × 30 mm × 30 mm (L × W × H) were prepared using the optimum 3D printable geopolymer mix (Figure 3b). The first layer was extruded, and the second layer was then extruded on top of the first layer after 2 min (the delay time or print-time interval between the layers).

All printed filaments were placed in a sealed container to prevent excessive moisture loss and kept in the laboratory environment at ambient temperature (23 °C ± 3 °C) until the testing day. The testing day for part I was 3 days after printing, while for part II it was 7 and 28 days after printing.

### 3.3. Testing

#### 3.3.1. Workability

The workability of fresh mixtures was measured by conducting a mini-slump test in accordance with ASTM C1437 [31].

#### 3.3.2. Extrudability

The method proposed by Le et al. [11] was followed in this paper to evaluate the extrudability of each geopolymer mixture. In this regard, for each mixture, four 250 mm long single-layer filaments were extruded. A mixture was considered to have “acceptable” extrudability if all four filaments could be extruded through the nozzle successfully without any blockage, tearing, segregation or bleeding.

#### 3.3.3. Shape Retention Ability

The shape retention ratio (SRR) was defined in this paper to quantify the shape retention ability of each mixture. The SRR can be calculated by the following equation:

(1) $$\mathrm{SRR}=\;\frac{W_{\mathrm{Nozzle}}}{W_{\mathrm{Filament}}}$$

where W_Filament_ is the bottom width of the extruded filament and W_Nozzle_ is the width of the nozzle opening. The higher the SRR, the better the shape retention ability of the mixture. As mentioned in Section 3.2, W_Nozzle_ was equal to 30 mm in this paper. For each mix, specimens measuring 60 mm × 30 mm × 15 mm (L × W × H) were sawn from the 250 mm × 30 mm × 15 mm (L × W × H) printed filaments. For each mix, the W_Filament_ of at least six 60 mm × 30 mm × 15 mm specimens was measured using a digital Vernier calliper with an accuracy of up to 0.01 mm. The average SRR and the standard deviation values were calculated.

#### 3.3.4. Compressive Strength

**Figure 4:**
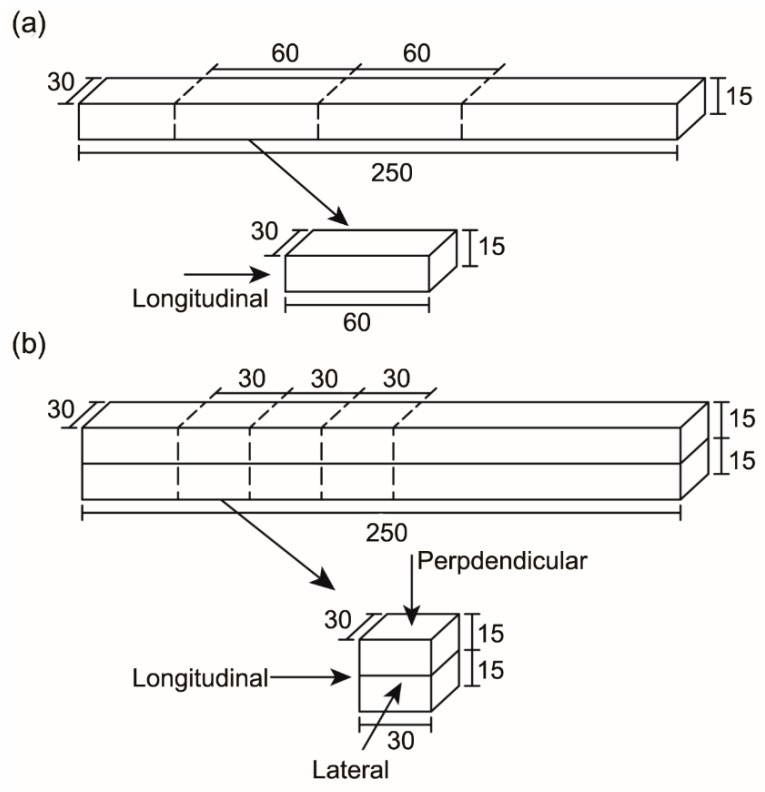
Dimensions and testing directions for the compression test of (**a**) the 3D printed one-layer specimen and (**b**) the 3D printed two-layer specimen.

In part I, specimens with dimensions of 60 mm × 30 mm × 15 mm (L × W × H) were sawn from the 250 mm × 30 mm × 15 mm (L × W × H) printed filaments and tested in the longitudinal direction only (Figure 4a). The average value of six tested specimens was reported as the compressive strength of the corresponding mix. In part II, 30 mm cube specimens were sawn from the 250 mm × 30 mm × 30 mm (L × W × H) printed filaments and tested in one of the three directions: Longitudinal, lateral or perpendicular (Figure 4b). The average value of the three tested specimens was reported as the compressive strength of the corresponding mix. A loading rate of 0.33 MPa/s, using uniaxial compression, was applied to all specimens. 

#### 3.3.5. Flexural Strength

**Figure 5:**
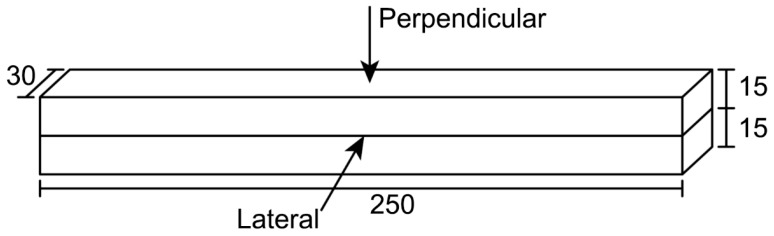
Testing directions for the flexural test of the two-layer optimum 3D printed geopolymer mix.

In part II, the flexural strength of the optimum mixture was tested in different loading directions. Three filaments with dimensions of 250 mm × 30 mm × 30 mm (L × W × H) for each of the lateral and perpendicular directions (six samples in total) were printed (Figure 5). A three-point bending test with a 150 mm span length was adopted. All specimens were tested under displacement control at the rate of 1.0 mm/min. It is pointing out that all surfaces of the 3D printed specimens were ground to have a flat and smooth surface before conducting the compression and flexural tests.

#### 3.3.6. Interlayer Bond Strength

**Figure 6:**
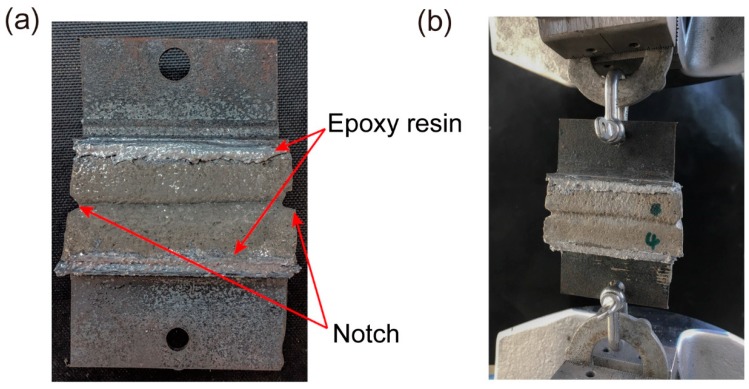
(**a**) Specimen prepared for interlayer bond test. (**b**) Test setup for interlayer bond tests.

Another task of part II was the measurement of the interlayer bond strength of the selected optimum mixture from part I. Specimens with dimensions of 50 mm × 30 mm × 30 mm (L × W × H) were sawn from the 250 mm × 30 mm × 30 mm (L × W × H) printed filaments. The average value of five tested samples was considered as the bond strength of the optimum mix. To measure the bond strength, two metallic T-sections were connected to the bottom and top of the sawn samples using epoxy glue. To ensure that the failure of the samples occurred at the interface, a small notch was created at either side of the interface (Figure 6a). A displacement rate of 1 mm/min was applied to all specimens using uniaxial tension (Figure 6b). To avoid any eccentricity, extra attention was taken to have specimens aligned in the tensile machine.

## 4. Results and Discussions

### 4.1. Results of Part I

#### 4.1.1. Extrudability

**Figure 7:**
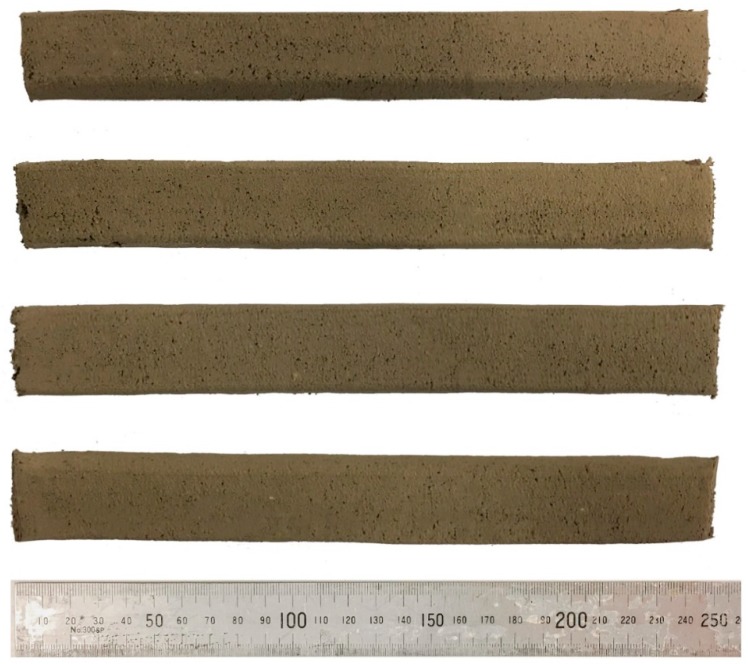
Four 250 mm printed filaments of the “Na-N-2.5” mixture.

According to the results, no blockage, tearing, segregation or bleeding was observed during the extrusion process of all mixtures. Therefore, it can be concluded that all mixtures investigated in this paper exhibited “acceptable” extrudability. For example, Figure 7 depicts four printed filaments from the “Na-N-2.5” mixture, showing the “acceptable” extrudability of this mixture. Based on the results obtained, the investigated parameters showed insignificant influence on the extrudability of the tested fresh mixes. This is because the dosage of admixtures (i.e., borax and CMC) were adjusted in each mixture to obtain useful rheological properties appropriate for 3DCP. It should be noted that the extrudability of a 3D printable cementitious material is influenced by several factors, including the printing parameters (e.g., size of nozzle, type of extruder and extrusion rate), mixture proportions and rheological properties of the mixture (e.g., viscosity and yield stress) [11,32,33].

#### 4.1.2. Workability

**Figure 8:**
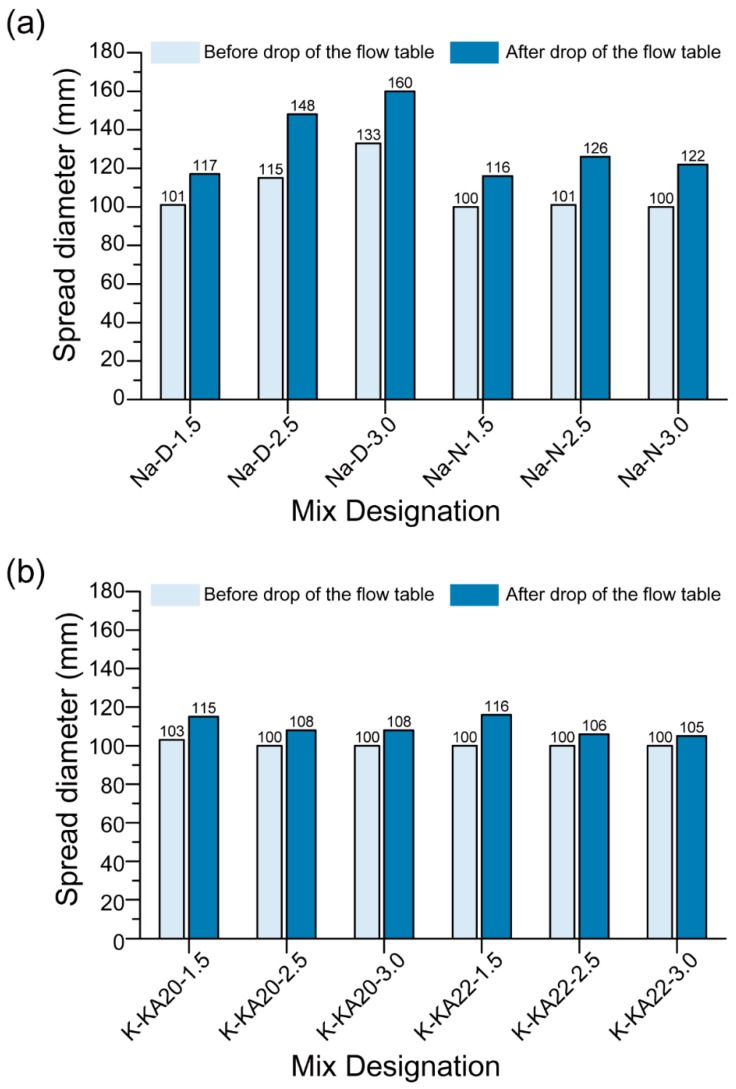
Workability test results of the 3D printable geopolymers with (**a**) Na-based activators and (**b**) K-based activators.

Figure 8 shows the average workability of the fresh mixtures before and after the drop of the flow table. The average spread dimeter of all mixtures, except “Na-D-2.5” and “Na-D-3.0”, before the drop of the flow table was almost 100 mm, i.e., equal to the diameter of the bottom of the mini-slump cone. This indicates all fresh mixtures except “Na-D-2.5” and “Na-D-3.0” had zero-slump. Having a mixture with zero-slump is beneficial for extrusion-based 3DCP mixtures as it helps the filament to keep its shape when extruded. It should be noted that although the “Na-D-2.5” and “Na-D-3.0” mixtures contained the highest amount of CMC and had the lowest W/GP-solids, the mixtures still exhibited the highest spread diameter, both before and after the drop of the flow table, which makes them unsuitable for extrusion-based 3DCP. This is because of the high viscosity of D grade Na_2_SiO_3_ when the W/GP-solids decrease. The resultant solution has an insufficient ability to wet all solid particles and hence proper mixing does not occur. Therefore, the initial setting time for the mix increases considerably and the spread diameter of the mix becomes the highest out of all mixes.

For a constant mass ratio of SS to HS of 1.5, the average spread diameter of the Na-based geopolymer mixture after the drop of the flow table was comparable to that of the corresponding K-based geopolymer mixture. Nevertheless, for constant mass ratios of SS and HS of 2.5 and 3.0, respectively, the average spread diameter of the Na-based geopolymer mixture after the drop of the flow table was 13–52% higher than that of the corresponding K-based geopolymer mixture, depending on the type of SS used. A previous study showed that slump is affected by the yield stress of the paste that is used [34]. Therefore, the higher workability of the Na-based geopolymer mixtures than the K-based geopolymer mixtures indicates lower yield stress. In the K-based geopolymer mixtures, for a constant mass ratio of SS to HS, regardless of the type of K-silicate solution used, the average spread diameter after the drop of the flow table was comparable. In general, it can be concluded that the type of HS, the type of SS and the mass ratio of SS to HS had considerable effects on the workability of the fresh geopolymer mixtures investigated in this paper.

#### 4.1.3. Shape Retention Ability

**Figure 9:**
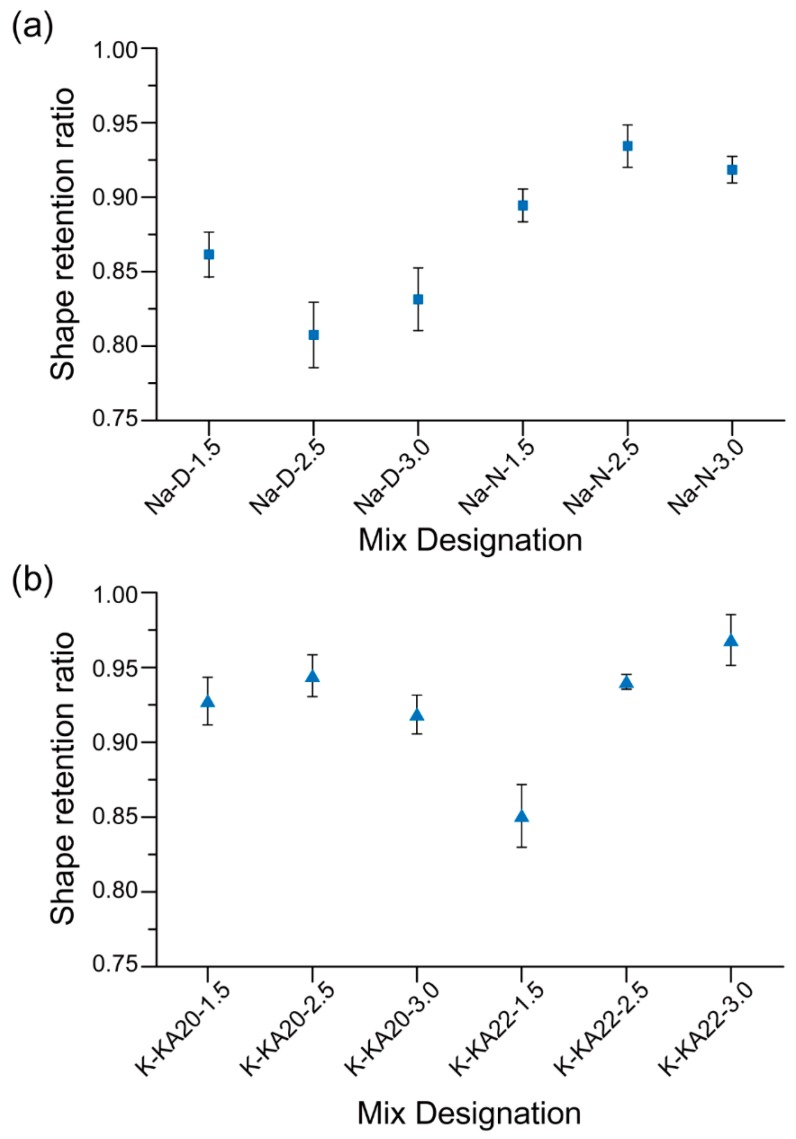
Shape retention ratios of 3D printable geopolymers with (**a**) Na-based activators and (**b**) K-based activators.

Figure 9 shows the SRRs of the 3D printable geopolymer mixtures. Among the Na-based geopolymers, the mixtures made of D grade Na_2_SiO_3_ solution contained a higher amount of CMC powder (Table 3), however, the SRRs of these mixtures were lower than that of the mixtures made with the N grade Na_2_SiO_3_ solution. This is true regardless of the SS/HS mass ratio. This result is consistent with the workability results (Figure 8a), where the “Na-D-2.5” and “Na-D-3.0” mixtures had the highest spread diameter both before and after the dropping of the flow table, indicating the poor shape retention ability of these mixtures.

Among the geopolymers made of N grade Na_2_SiO_3_ solution, the “Na-N-2.5” mixture exhibited the highest mean SRR, and thereby the highest shape retention ability. The higher SRR of the “Na-N-2.5” mixture than that of the “Na-N-1.5” mixture is due to its lower W/GP-solids and higher CMC content. The SRR of the “Na-N-3.0” mixture was lower than that of the “Na-N-2.5” mixture, which may be due to its lower CMC content.

As can be seen in Figure 9b, the SRR of the K-based geopolymers significantly depended on the type of SS and mass ratio of SS/HS. The “K-KA22-1.5” mixture exhibited the lowest SRR and thereby the lowest shape retention ability. This may be because this mixture had the highest W/GP-solids among the K-based mixtures (Table 3). 

#### 4.1.4. Compressive Strength

**Figure 10:**
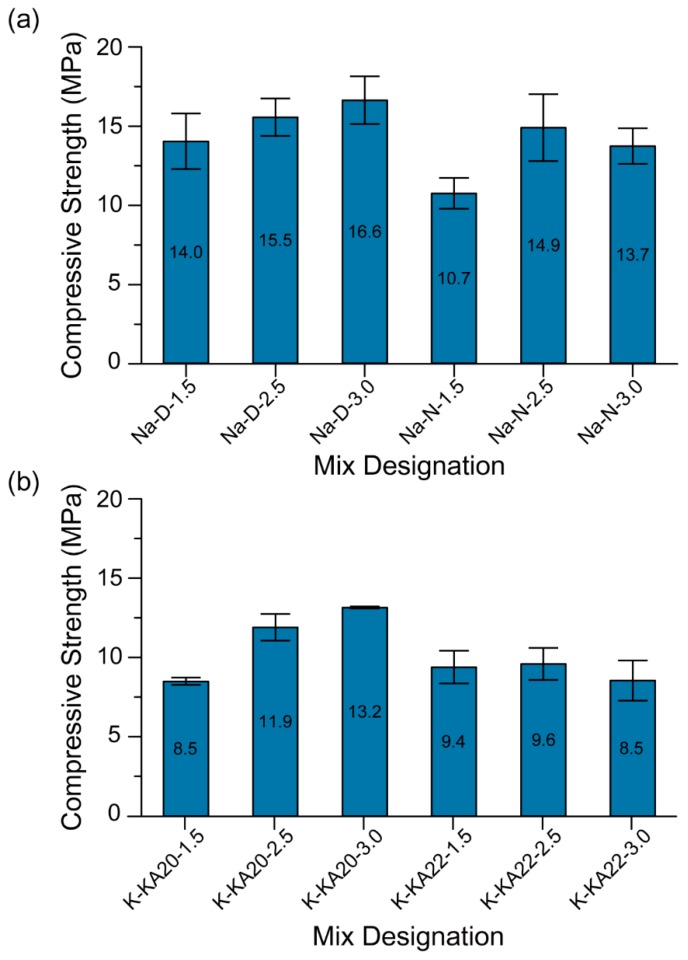
Compressive strength of 3D printed geopolymers with (**a**) Na-based activators and (**b**) K-based activators.

The 3-day compressive strength of the 3D printable geopolymer mixtures is shown in Figure 10. As mentioned in Section 3.3.4, the specimens tested in this section consisted of a single layer only, tested in the longitudinal direction. Regardless of the type of SS and HS, the mean compressive strength of printed geopolymers increases when raising the SS/HS mass ratio from 1.5 to 2.5. Nonetheless, the type of HS and SS are the factors that determine the rate of compressive strength increase. This is because the increase of the SS/HS mass ratio not only reduces the W/GP-solids (Table 3), but also increases the soluble silicate content in the geopolymeric system. As a result, the geopolymerisation reaction and hence compressive strength improves [35,36].

In addition, the increase in the SS/HS mass ratio from 2.5 to 3.0 slightly increased the mean compressive strength of printed geopolymers made of D grade Na_2_SiO_3_ solution (SiO_2_/Na_2_O = 2.00) or KASIL 2040 grade K_2_SiO_3_ solution (SiO_2_/K_2_O = 2.02). However, the increase in the SS/HS mass ratio from 2.5 to 3.0 slightly reduced the mean compressive strength of printed geopolymers made from the N grade Na_2_SiO_3_ solution (SiO_2_/Na_2_O = 3.22) or the KASIL 2236 grade K_2_SiO_3_ solution (SiO_2_/K_2_O = 2.22). This is because of the use of SS with a higher SiO_2_/M_2_O ratio (M = Na or K). The slight increase or decrease in the compressive strength of printed geopolymers due to the increase of the SS/HS mass ratio from 2.5 to 3.0 depended on the SiO_2_/M_2_O ratio of SS. Previous studies have reported that the increase in compressive strength of geopolymers directly relates to the content of silicon. However, there is an optimum silicon content where further increase has a slight or negative impact in terms of increasing the compressive strength [36,37].

As can be seen in Figure 10, geopolymers with Na-based activators have higher compressive strength than K-based ones. This result is valid for all samples, regardless of the type of SS and the SS/HS mass ratio. Palomo et al. [38] have reported the same results for the conventionally mould-casted geopolymers. Among the Na-based geopolymers, the mixtures containing the D grade Na_2_SiO_3_ solution exhibited higher compressive strength than that of the mixtures containing the N grade Na_2_SiO_3_ solution. This is true regardless of the SS/HS mass ratio. However, as mentioned in Section 4.1.3 (Figure 9a), the shape retention ability of the mixtures made of the D grade Na_2_SiO_3_ solution was lower than that of the mixtures made from the N grade Na_2_SiO_3_ solution. Therefore, the “Na-N-2.5” mixture was selected as the optimum mixture suitable for 3D printing because it exhibited the highest compressive strength and shape retention ability among the mixtures made from the N grade Na_2_SiO_3_ solution.

### 4.2. Results of Part II

#### 4.2.1. Compressive Strength

**Figure 11:**
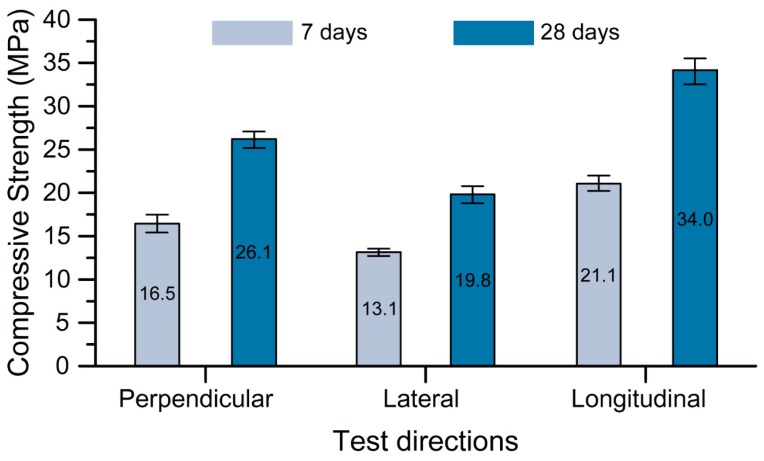
Compressive strength of Na-N-2.5 (the optimum 3D printable mixture) in different directions.

Figure 11 presents the 7-day and 28-day compressive strengths of the Na-N-2.5 mix (the optimum 3D printable mixture) in the tested loading directions. Similar to other 3D printed concretes, the optimum mix exhibited anisotropic behaviour with respect to the loading directions [11,22,39]. Figure 11 shows that the longitudinal and lateral loading directions have the highest and the lowest compressive strength at any age, respectively. This agrees with the results obtained by Sanjayan et al. [39] for printable OPC concrete and Panda et al. [40] and Nematollahi et al. [41] for printable geopolymer concrete. High pressure applies to the fresh material during extrusion in the print direction and hence it is anticipated to have the highest strength in longitudinal direction [39,40]. On the other hand, no pressure is applied in the lateral direction, and the printed material expands and settles freely in this direction, therefore, it shows the lowest strength in this direction [39]. The fresh material in the perpendicular direction experiences some pressure because of its own weight and hence it has a strength between the materials printed in the other two directions.

As expected, the samples cured for 28 days had higher strengths than those cured for 7 days. Deb et al. [42] reported similar results for conventionally mould-casted geopolymers. It is interesting that the loading direction was effective on this increase, where the rate of increase in the longitudinal, perpendicular and lateral directions was 61%, 58% and 51%, respectively.

#### 4.2.2. Flexural Strength

**Figure 12:**
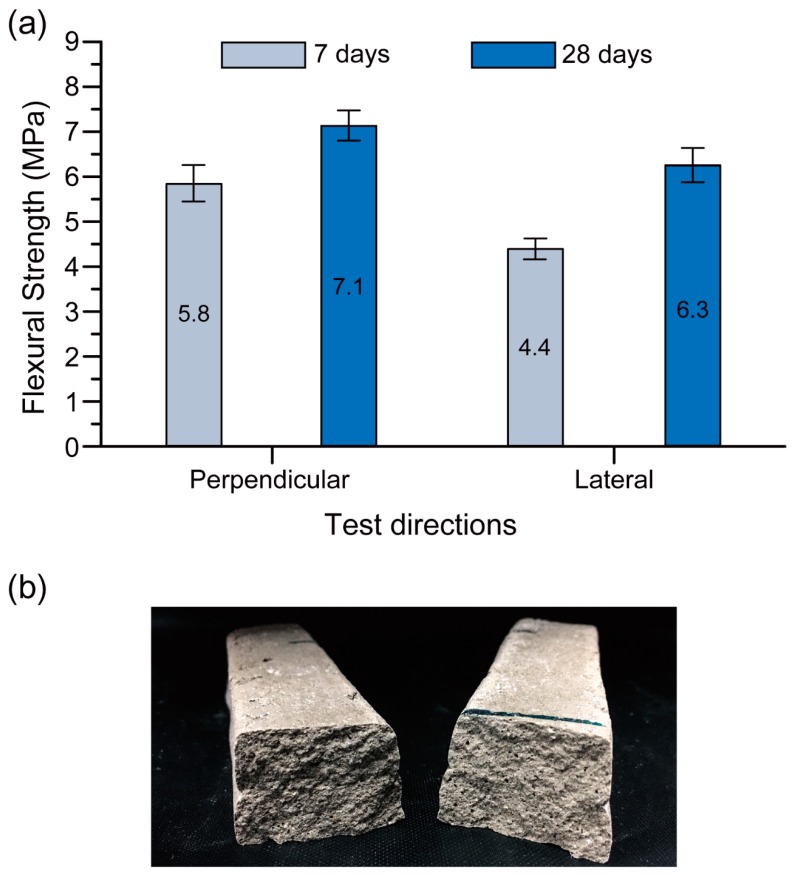
(**a**) Flexural strength of Na-N-2.5 (the optimum 3D printable mixture) in different directions. (**b**) Side view of the fractured surfaces.

Figure 12a shows the 7-day and 28-day flexural strengths of the Na-N-2.5 mix (the optimum 3D printable mixture) in the tested loading directions. Similar to the compressive strength results, anisotropic behaviour was observed for flexural strength testing in the different load directions [11,22,39]. This behaviour could be considered as an inherent characteristic which is observable in additive manufacturing processes and layered composites [43]. The failure observed in the flexural testing was a tensile failure rather than a shear failure of the interface. The seamless interface between layers is noticeable in Figure 12b, supporting this statement.

Figure 12a shows that the testing of the printed mix under flexural load in the perpendicular direction exhibited higher strength than in the lateral direction for any age of curing. This agrees with the results obtained by Sanjayan et al. [39] for printable OPC concrete and Panda et al. [40] for printable geopolymer concrete. Because of the weight of the top layer during printing, the bottom layer is probably well compacted and hence the flexural strength of the sample in the perpendicular load direction is higher than the lateral direction [39].

The flexural strength of the Na-N-2.5 mix was also significantly higher at 28 days than that at 7 days, owing to the longer age of curing. The loading direction was effective on this increase where the rate of increase in lateral and perpendicular directions was 43% and 22% respectively.

The compressive strength of the material tested in the perpendicular direction after 28 days of curing was 32% higher than the same material tested in the lateral direction. This difference for the flexural strength test was only 13%. This result indicates that the anisotropic behaviour in the compression test of the optimum 3D printed geopolymer mix was more pronounced than in the flexural test.

#### 4.2.3. Interlayer Bond Strength

**Figure 13:**
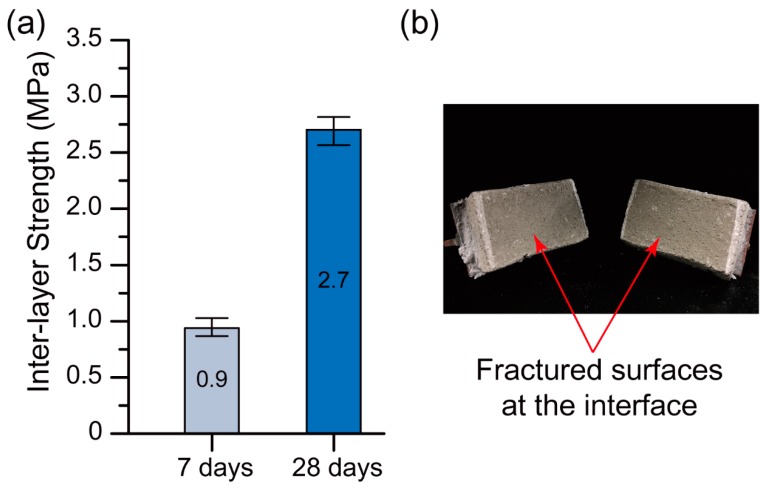
(**a**) Interlayer bond strength of Na-N-2.5 (the optimum 3D printable mixture). (**b**) Failure mode of a tested specimen.

Figure 13a presents the 7-day and 28-day interlayer bond strengths of the Na-N-2.5 mix (the optimum 3D printable mixture). The mean interlayer bond strength at 28 days was exactly three times higher than that at 7 days. It should be noted that the 7-day interlayer bond strength (0.9 MPa) obtained was still adequate to avoid interfacial shear failure. The failure mode in the flexural test, which was tensile, not shear, confirms this statement. Figure 12b supports the high interlayer bond strength obtained because there was not any noticeable layer interface on the fractured surfaces of the printed specimen. As shown in Figure 13b, regardless of the testing age, the failure of all interlayer bond strength samples happened at the bottom and top layer interfaces.

The 28-day interlayer bond strength obtained was almost two times higher than that obtained in a previous study [22], where the strength was 1.4 MPa as compared to the 2.7 MPa obtained. This reveals that optimising material ingredients is of high importance.

## 5. Conclusions

An optimum 3D printable geopolymer mixture cured at ambient temperature was developed in this paper by optimizing the geopolymer mixture parameters (including the type of hydroxide solutions (HS), the type of silicate solutions (SS) and the SS/HS mass ratio) across multiple performance criteria (including workability, extrudability, shape retention ability and compressive strength). According to the testing direction, the optimum mixture exhibited a 28-day compressive strength of 19.8–34.0 MPa and a flexural strength of 6.3–7.1 MPa. Additionally, a 28-day interlayer bond strength of 2.7 MPa was achieved. From the obtained results, the following conclusions can be made:

1. The parameters investigated had insignificant impact on the extrudability of the mixtures, as no blockage, tearing, segregation, or bleeding was observed during the extrusion process of all mixtures. This is because the dosage of admixtures (i.e., viscosity modifying agent and retarder) were adjusted in each mixture to obtain beneficial rheological properties appropriate for concrete 3D printing, based on the extrusion method.
2. The mixture parameters investigated had considerable effects on the workability of the fresh printable geopolymers. In general, the sodium (Na)-based geopolymers had higher workability than the potassium (K)-based ones, which indicates the lower yield stress of the Na-based geopolymers than that of the K-based geopolymers.
3. The shape retention ability of the printable geopolymers made from the Na_2_SiO_3_ solution with SiO_2_/Na_2_O of 3.22 (N grade) was higher than that of the mixtures made from the Na_2_SiO_3_ solution with SiO_2_/Na_2_O of 2.00 (D grade) for all SS/HS mass ratios.
4. Na-based printable geopolymers had higher compressive strengths than K-based ones for all types of SS and SS/HS mass ratios.
5. The compressive strengths of the optimum printable mixture for the printed materials tested in the longitudinal, perpendicular and lateral directions were 34.0, 26.1 and 19.8 MPa, respectively.
6. The flexural strengths of the optimum printable mixture for the printed materials tested in the perpendicular and lateral directions were 7.10 and 6.30 MPa, respectively.

## Figures and Tables

**Table 1:** Chemical composition of the slag and fly ash (wt.%).

Chemical	Component	
	Slag	Fly Ash
Al_2_O_3_	12.4	25.6
SiO_2_	32.8	51.1
CaO	44.6	4.30
Fe_2_O_3_	0.54	12.5
K_2_O	0.33	0.70
MgO	5.15	1.45
Na_2_O	0.22	0.77
P_2_O_5_	0.88	0.01
TiO_2_	0.51	1.32
MnO	0.15	0.37
SO_3_	4.26	0.24
L.O.I. ^1^	0.09	0.57

^1^ Loss on ignition.

**Table 2:** Specifications of the silicate solutions (SSs).

Type of SS	SiO_2_/M_2_O *	SiO_2_ (wt.%)	M_2_O * (wt.%)	H_2_O (wt.%)	Viscosity ^1^ (cps)	Density ^1^ (g/cc)
D Grade Na_2_SiO_3_	2.00	29.4	14.7	55.9	250–450	1.52
N Grade Na_2_SiO_3_	3.22	28.7	8.9	62.4	100–300	1.38
KASIL 2040 Grade K_2_SiO_3_	2.02	26.7	13.3	60.0	100–300	1.39
KASIL 2236 Grade K_2_SiO_3_	2.22	24.5	11.0	64.5	80–120	1.33

* M in M_2_O refers to Na or K; ^1^ The viscosity and density values are reported at 20 °C.

**Table 3:** The mixture proportions of 3D printable geopolymers.

Mix Designation	Source Materials	Activator		Sand		Borax ^g^	CMC ^h^	W/GP-Solids
		HS	SS	CS	FS			
Na-D-1.5	1.000	0.160 ^a^	0.240 ^c^	1.000	0.500	0.002	0.015	0.220
Na-D-2.5	1.000	0.114 ^a^	0.286 ^c^	1.000	0.500	0.002	0.020	0.211
Na-D-3.0	1.000	0.100 ^a^	0.300 ^c^	1.000	0.500	0.002	0.020	0.208
Na-N-1.5	1.000	0.160 ^a^	0.240 ^d^	1.000	0.500	0.002	0.011	0.236
Na-N-2.5	1.000	0.114 ^a^	0.286 ^d^	1.000	0.500	0.002	0.013	0.231
Na-N-3.0	1.000	0.100 ^a^	0.300 ^d^	1.000	0.500	0.002	0.010	0.229
K-KA20-1.5	1.000	0.160 ^b^	0.240 ^e^	1.000	0.500	0.002	0.010	0.222
K-KA20-2.5	1.000	0.114 ^b^	0.286 ^e^	1.000	0.500	0.004	0.009	0.218
K-KA20-3.0	1.000	0.100 ^b^	0.300 ^e^	1.000	0.500	0.005	0.007	0.216
K-KA22-1.5	1.000	0.160 ^b^	0.240 ^f^	1.000	0.500	0.005	0.008	0.234
K-KA22-2.5	1.000	0.114 ^b^	0.286 ^f^	1.000	0.500	0.005	0.006	0.232
K-KA22-3.0	1.000	0.100 ^b^	0.300 ^f^	1.000	0.500	0.005	0.004	0.231

Note: All numbers are mass ratios of the source material (fly ash and slag) weights. ^a^ The 8.0 M NaOH solution. ^b^ The 8.0 M KOH solution. ^c^ The D grade Na_2_SiO_3_ solution. ^d^ The N grade Na_2_SiO_3_ solution. ^e^ The KASIL 2040 grade K_2_SiO_3_ solution. ^f^ The KASIL 2236 grade K_2_SiO_3_ solution. ^g^ Used as the retarder. ^f^ Used as the viscosity modifying agent.
